# Involvement of neuronal IL-1β in acquired brain lesions in a rat model of neonatal encephalopathy

**DOI:** 10.1186/1742-2094-10-110

**Published:** 2013-09-05

**Authors:** Alexandre Savard, Karine Lavoie, Marie-Elsa Brochu, Djordje Grbic, Martin Lepage, Denis Gris, Guillaume Sebire

**Affiliations:** 1 Laboratoire de Neurologie Pédiatrique, Université de Sherbrooke, 3001 12e Avenue Nord, J1H 5N4 Sherbrooke, Québec, Canada; 2 Département de Pédiatrie, Université de Sherbrooke, Sherbrooke, Québec, Canada; 3 Département de Médecine Nucléaire et Radiobiologie, Université de Sherbrooke, Sherbrooke, Québec, Canada

**Keywords:** Hypoxia-ischemia, Term newborn, Pathogen exposure, Inflammation

## Abstract

**Background:**

Infection-inflammation combined with hypoxia-ischemia (HI) is the most prevalent pathological scenario involved in perinatal brain damage leading to life-long neurological disabilities. Following lipopolysaccharide (LPS) and/or HI aggression, different patterns of inflammatory responses have been uncovered according to the brain differentiation stage. In fact, LPS pre-exposure has been reported to aggravate HI brain lesions in post-natal day 1 (P1) and P7 rat models that are respectively equivalent - in terms of brain development - to early and late human preterm newborns. However, little is known about the innate immune response in LPS plus HI-induced lesions of the full-term newborn forebrain and the associated neuropathological and neurobehavioral outcomes.

**Methods:**

An original preclinical rat model has been previously documented for the innate neuroimmune response at different post-natal ages. It was used in the present study to investigate the neuroinflammatory mechanisms that underline neurological impairments after pathogen-induced inflammation and HI in term newborns.

**Results:**

LPS and HI exerted a synergistic detrimental effect on rat brain. Their effect led to a peculiar pattern of parasagittal cortical-subcortical infarcts mimicking those in the human full-term newborn with subsequent severe neurodevelopmental impairments. An increased IL-1β response in neocortical and basal gray neurons was demonstrated at 4 h after LPS + HI-exposure and preceded other neuroinflammatory responses such as microglial and astroglial cell activation. Neurological deficits were observed during the acute phase of injury followed by a recovery, then by a delayed onset of profound motor behavior impairment, reminiscent of the delayed clinical onset of motor system impairments observed in humans. Interleukin-1 receptor antagonist (IL-1ra) reduced the extent of brain lesions confirming the involvement of IL-1β response in their pathophysiology.

**Conclusion:**

In rat pups at a neurodevelopmental age corresponding to full-term human newborns, a systemic pre-exposure to a pathogen component amplified HI-induced mortality and morbidities that are relevant to human pathology. Neuronal cells were the first cells to produce IL-1β in LPS + HI-exposed full-term brains. Such IL-1β production might be responsible for neuronal self-injuries via well-described neurotoxic mechanisms such as IL-1β-induced nitric oxide production, or IL-1β-dependent exacerbation of excitotoxic damage.

## Introduction

Hypoxia-ischemia (HI) and/or infection-inflammation are the principal risk factors of perinatal cerebral injuries in both term and preterm human newborns [1-5]. However, clinical and pathological features of human perinatal brain lesions display striking variations according to the gestational ages [2]. Variations of brain damage according to gestational age have been also reported in newborn rodents exposed to HI [6,7]. In fact, distinct neuropathological signatures have been noticed depending on HI timing. HI exposure of rats at post-natal day 1 (P1) corresponding to the human early preterm stage of brain development results in multifocal white-matter lesions [6]. In contrast, HI exposure of rats at P7 or P12 corresponding respectively to the late preterm and full-term human brain stages of development results in severe parasagittal cortico-subcortical infarcts [6]. Recent human epidemiological and experimental studies highlighted that HI per se was not involved as frequently as thought in perinatal brain damage [1,3]. Infection-inflammation complicated by a transient HI is one of the most common physiopathological scenarios encountered in human perinatal brain insults and subsequent neonatal encephalopathy, leading to cerebral palsy [1,8]. Such combination of infection/inflammation and HI has been experimentally reproduced in rodents at neurodevelopmental stages equivalent to early and late preterm human neonates (rat pups at P1 and P7) [9-11]. It has also recently been reproduced in rats at a neurodevelopmental stage (P12) corresponding to full-term human neonates and profound differences have been shown between patterns of innate immune responses at P1 and P12 [12]. In the present study, our aim was to further characterize the neuroinflammatory mechanism that underlines neurological impairments after pathogen-induced inflammation and HI in term newborns.

## Materials and methods

### P12 rat model

The preclinical model was slightly modified from the protocol recently published by our group [12]. Briefly, Lewis dams were obtained from Charles River Laboratories (Saint-Constant, QC, Canada) at gestational day 16 (G16). They were kept at 20°C with a 12-h day/12-h night cycle, had unlimited access to food and water, and gave birth naturally. Pups were left untouched until P12 when they were given a single intraperitoneal injection of lipopolysaccharide (LPS) (200 μg/kg diluted in 50 μL of saline) or saline. Four hours after the LPS or saline injection, ischemia was induced by a permanent ligature of the right common carotid artery under isoflurane. A heating mat was used to maintain the pups' temperature at 37°C. A control group (Ctl) underwent no surgery or sham surgery, during which the common carotid artery was exposed but not ligated. Pups were returned to their dams for 30 minutes before being put in a hypoxia chamber with 8% oxygen at 37°C for 1.5 h or 3 h ± 30 minutes. Pups from each litter were randomized into different experimental groups independently of their sex and weight. The end of hypoxia was referred to 0 h. Pups were sacrificed at 4 h (P12), 24 h (P13), 48 h (P14), and 8 days (P20) post-HI. All the experiments were performed with the full approval of the Comité d’éthique de la Faculté de Médecine de l’Université de Sherbrooke.

### Mechanistic experiments testing the role of IL-1β

IL-1 receptor antagonist (IL-1ra) was used at a concentration of 200 mg/kg. This dose has already been demonstrated to be the most successful in an adult model of stroke [13]. The first injection was given 30 minutes before the LPS injection and five further injections were given every 12 h thereafter.

### Histology

Forebrains were fixed in 4% paraformaldehyde (PFA) at room temperature, paraffin-embedded, and cut into 5-μm slices using a microtome, for histological studies. H&E staining was used to visualize and quantify brain lesions. Image J analysis software (National Institutes of Health (NIH) Image, http://rsbweb.nih.gov/nih-image/) was used to measure the surface of the right hemisphere on coronal sections located at the epicenter of the infarct (Bregma −1.00). The surfaces of the right hemisphere of LPS- and/or HI-exposed rats were then compared with those of the Ctl.

### Immunohistochemistry (IHC) and immunofluorescence (IF)

IHC and IF were performed as previously described [9]. The antibodies used are detailed in Table 1. IF slides were mounted using a 4',6-diamidino-2-phenylindole (DAPI)-containing medium (Invitrogen, Burlington, ON, Canada). Negative controls consisted of an additional set of sections treated similarly but without the primary antibody. Counting of labeled cells was performed using the Image J analysis software (NIH Image, http://rsbweb.nih.gov/nih-image/).

**Table 1:** List and features of antibodies

**Antibody**	**Company – reference number**	**Dilution**
Anti-DIG-AP	Roche - 11093274910	1:3,000
Anti-NeuN	Millipore – MAB377	1:100
Anti-Iba-1	Abcam - ab15690	1:200
Anti-GFAP	Millipore - AB5541	1:500
Anti-PMN	Cedarlane - CLAD51140	1:200
Anti-IL-1β	Serotec - AAR15G	1:250
Anti-TNF-α	Millipore - AB1837P	1:250
Anti-rabbit-HRP	Serotec - STAR54	1:100
Anti-rabbit-Alexa Fluor conjugated	Invitrogen - A11012	1:500
Anti-mouse-Alexa Fluor conjugated	Invitrogen - A11005	1:500
Anti-chicken-Alexa Fluor conjugated	Invitrogen - A11039	1:500

### In situ hybridization

The expression and localization of mRNA encoding for IL-1β was detected on brain sections using dioxigenin-UTP(DIG)-labeled riboprobes. The IL-1β DNA template was amplified using primers with specific restriction enzymes: IL-1β Bam HI forward 5’- AGT CCT GGA TCC ATG GCA ACT GTC CCT GAA CT -3’ and IL-1β EcoRI reverse 5’- GGC CGC GAA TTC AGC TCA TGG AGA ATA CCA CT-3’. DIG-labeled single-stranded RNA was transcribed from commercially available plasmid vectors following the manufacturer instructions (Roche, Quebec, QC, Canada). The in situ hybridization protocol was provided by Dr Jasna Kriz [14]. Briefly, slides were dried, post-fixed in 4% PFA and digested by proteinase K, after which the brain sections were rinsed in water and in a solution of 0.1 M triethanolamine (TEA, pH 8.0) and acetylated in 0.25% acetic anhydride in 0.1 M TEA. The hybridization of the brains sections by the riboprobe was done overnight at 72°C. Slides were rinsed in standard saline citrate (1XSSC: 0.15 M NaCl, 15 mM trisodium citrate buffer, pH 7.0). Slides were incubated with anti-DIG-AP antibodies overnight at 4°C. Slides were incubated with nitro blue tetrazolium chloride (NBT)/(5-bromo-4-chloro-1H-indol-3-yl) dihydrogen phosphate (BCIP) solution for color development at room temperature for 16 h. Slides were rinsed and mounted with mounting medium. The staining intensities of IL-1β mRNA were studied by colorimetric analysis performed with Image J software as previously described [12].

### Brain magnetic resonance imaging (MRI) of the brain

MRI was performed on anesthetized rats at P20 using a small-animal 7 T MRI system (Varian, Palo Alto, CA, USA) equipped with 205/120. Magnex gradient coils and a 40-mm radiofrequency volume coil. The animals’ vital signs and temperature were monitored throughout the MRI procedure (Small Animal Instruments, Stony Brook, NY, USA). T2-weighted respiration-gated images were acquired using a fast spin-echo pulse sequence (TR/TEeff: 2,000/48 ms, 8 echoes, field of view (FOV): 2.5 × 2.5 cm^2^, matrix: (256)^2^, NA: 8, 20 slices of 1.0 mm). Lesion volumes were calculated using ITK-snap [15] and a three-dimensional model using Owl developed by *plateforme d’analyse et de visualisation d’images* (PAVI) at the Université de Sherbrooke.

### ELISA

Protein extracts were prepared from the right hemisphere of the forebrain as previously described [12]. ELISA was performed on these protein extracts using ELISA Kits (R&D Systems, Minneapolis, MN, USA), as previously described [12].

### Animal behavioral tests

The circling behavioral test was analyzed using the open-field test apparatus. Rat pups at P13 and P14 were placed in the center of the open field and videotaped for 1.5 minutes. The movement of the pups was scored: 0 = absence of circling, 1 = partial circling, 2 = total circling. The open field test was used to determine spontaneous locomotor activity and exploratory behavior from P15 to P25 and from P100 to P120, as described previously [9]. Motor balance and agility were also analyzed by the rotarod test from P30 to P40, as previously described [9]. The elevated body swing test was used to determine long-term motor impairment from P100 to P120. The movement of the rats suspended by their tail was captured by a camera. We set the threshold at an angle of 90° for an efficient upswing. Latency before the first upswing, the side of the upswing, and the total number of upswings were recorded and compared under different experimental conditions. The turning-in-alley test was performed at P100-120 to assess a potential persisting shift of lateralization due to motor impairment. Rats were placed facing the end of a closed alley: the duration and the side of the turning were recorded.

### Data analysis

Data are presented as mean ± standard error of the mean (SEM). Comparisons between different conditions were performed using analysis of variance (ANOVA) with Newman-Keuls post-test or the unpaired *t*-test with the Welch correction. The statistical significance level was set at *P* ≤ 0.05. Mortality was analyzed with the *X*^2^ test. Data from saline-injected pups with sham surgery, or no surgery, as well as male and female data were combined because they were not significantly different.

## Results

### Mortality and weight loss induced by LPS and/or HI

**Figure 1:**
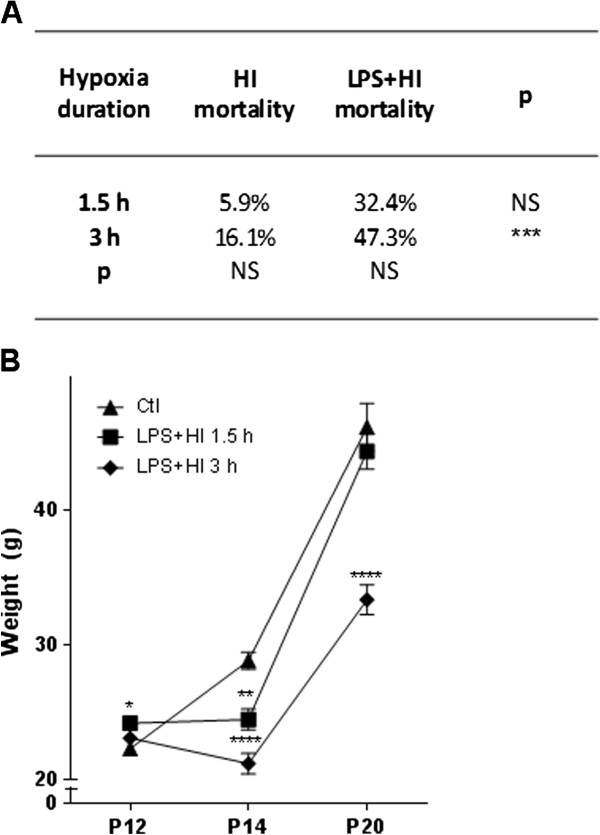
**Pup mortality during hypoxia and weight chart of the surviving pups. (A)** Mortality (%) according to hypoxia duration in lipopolysaccaride (LPS) ± hypoxia-ischemia (HI)-exposed pups (20 to 150 pups per condition). *χ*^2^ test: ^***^P ≤ 0.001. **(B)** Pup-weight follow up from post-natal day (P)12 to P20 (10 to 30 pups per condition and time point). Mean ± standard error of the mean; analysis of variance with Newman-Keuls post-test; ^*^P ≤0.05, ^**^P ≤ 0.01, ^****^P ≤ 0.0001. *h* hour, *g* gram, *NS* Not significant.

Combination of LPS plus HI stimulation induced the highest mortality, peaking after 3 h of hypoxia (Figure 1A). A transient lack of weight gain was observed under LPS + HI treatment after 1.5 h of hypoxia (Figure 1B).

### Neuropathological alterations induced by LPS and/or HI

**Figure 2:**
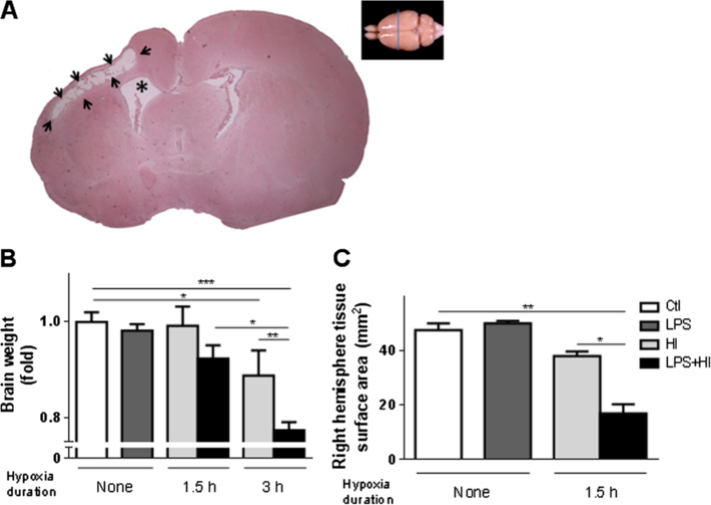
**Quantification of lipopolysaccharide (LPS) + hypoxia-ischemia (HI)-induced brain damage at post-natal day (P)20. (A)** Cavitary damage (arrows) and ventricle enlargement (^*^) within the right hemisphere ipsilateral to HI in LPS + HI-exposed pups. **(B)** Mean ± standard error of the mean forebrain weight loss (fold decrease compared to control (Ctl)), and **(C)** right hemisphere surface (mm^2^) after LPS ± HI-exposure (4 to 6 pups per experimental condition). Analysis of variance with Newman-Keuls post-test; ^*^P ≤ 0.05, ^**^P ≤ 0.01, ^***^P ≤ 0.001. *h* hour.

Brain damage induced by HI and by LPS + HI was ipsilateral to the right common carotid occlusion. In the right cerebral hemispheres from LPS + HI-exposed animals infarcted areas appeared as confluent microcystic or large cavitary lesions, significantly more extended compared to HI exposure alone (Figure 2A and [12]). Forebrain weights were significantly reduced at 8 days (P20) post-HI (0.89-fold after 3 h of hypoxia), and post-LPS plus HI (0.9-fold and 0.77-fold respectively after 1.5 h or 3 h of hypoxia), compared to Ctl. Maximal forebrain weight loss was found after LPS + HI exposure (Figure 2B) compared to HI or LPS exposure alone. The brains of LPS + HI-exposed pups also displayed a significantly reduced surface of the right hemisphere compared to other experimental conditions (Figure 2C). Brains exposed to LPS alone did not show any histological change compared to Ctl (data not shown).

**Figure 3:**
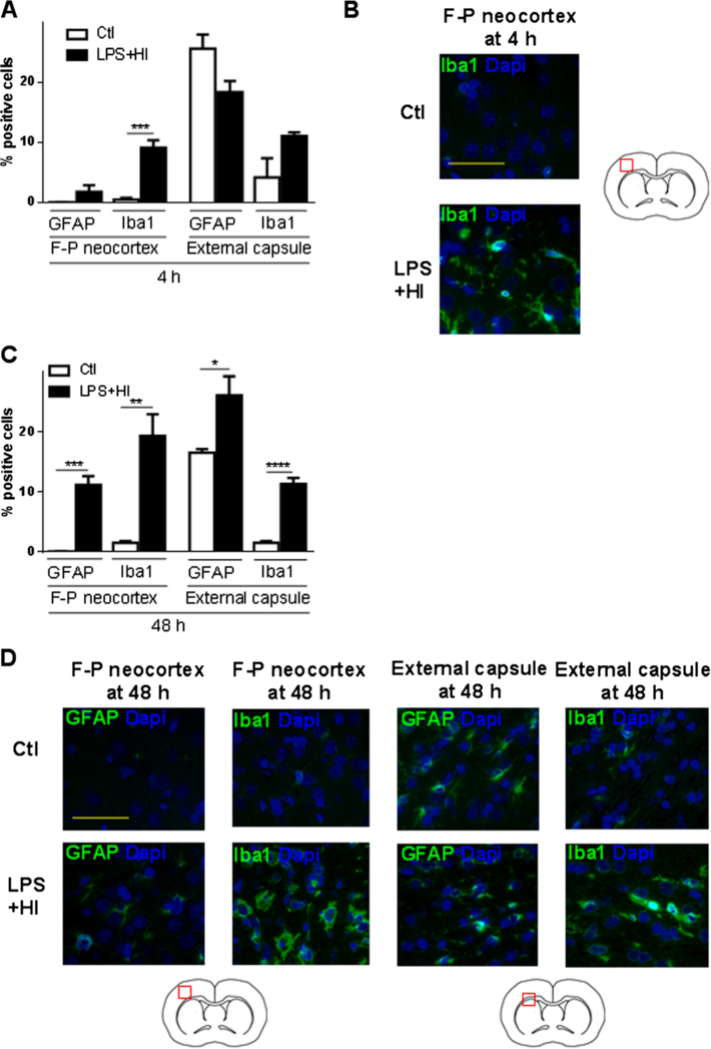
**Astrocyte and macrophage/microglia in the right cerebral hemisphere.** Percentages of macrophage/microglia (ionized calcium-binding adapter molecule, 1Iba-1+) and astrocyte (Glial fibrillary acidic protein (GFAP)+) after lipopolysaccharide (LPS) + hypoxia-ischemia (HI)-exposure versus control (Ctl) at 4 h **(A)** and 48 h **(C)**. **(B)** Immunofluorescence (IF) staining for Iba-1+ cells in the right fronto-parietal neocortex at 4 h. **(D)** IF staining for Iba-1+ and GFAP + cells in the right external capsule and fronto-parietal neocortex exposed to LPS plus HI versus Ctl (3 to 4 pups per experimental condition). Scale bar = 50 μm. Mean ± standard error of the mean; analysis of variance with Newman-Keuls post-test, ^*^P ≤ 0.05, ^**^P ≤ 0.01, ^***^P ≤ 0.001, ^****^P ≤ 0.0001. *h* hour, *F-P* Fronto-parietal.

**Figure 4:**
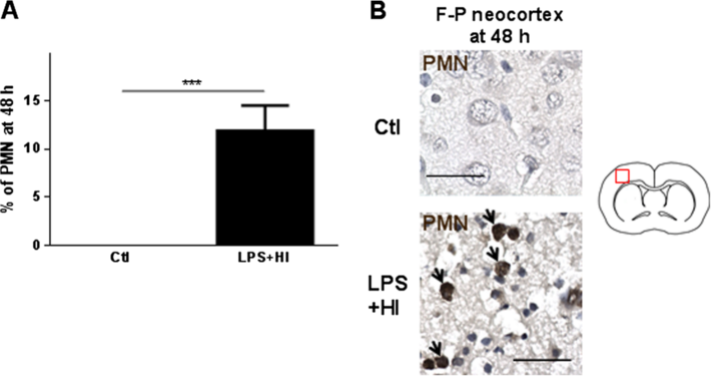
**Infiltration of polymorphonuclear neutrophils (PMN) in the right fronto-parietal neocortex exposed to lipopolysaccharide (LPS) plus hypoxia-ischemia (HI) at 48 hours. (A)** Mean ± standard error of the mean percentage of PMN infiltration after LPS + HI-exposure versus control (Ctl) at 48 h. **(B)** Immunohistochemical staining of PMN (arrows) in the fronto-parietal neocortex of LPS + HI-exposed rats versus Ctl, at 48 h (5 to 6 pups per condition). Scale bar = 50 μm. Unpaired *t*-test with Welch correction; ^***^P ≤ 0.001. *h* hour, *F-P* Fronto-parietal.

Given the high mortality induced by 3 h of hypoxia (Figure 1A), we decided to conduct our following experiments under 1.5 h of hypoxia. We further investigated the LPS + HI condition because, in contrast to HI alone, it has never been studied before at the P12 term-like developmental stage, except for comparing the overall cytokine and chemokine production within the brain, between P1 and P12 rats [12]. After LPS + HI, a rapid increase of ionized calcium-binding adapter molecule 1 (Iba-1) + microglia/macrophages was observed in the right neocortex compared to Ctl at 4 h (18-fold increase) and at 48 h (13-fold increase); Iba-1+ macrophage/microglia infiltration was delayed - appearing only at 48 h - in the underlying external capsule (8-fold increase) (Figure 3A-D) and in the right caudate putamen (data not shown). Glial fibrillary acidic protein (GFAP) + astrocyte significantly increased at 48 h (not at 4 h) in the right neocortex of LPS + HI-exposed pups (11-fold increase), the underlying external capsule (1.6-fold increase) and in the caudate putamen (data not shown) compared to Ctl (Figure 3A, C, D). Polymorphonuclear neutrophil (PMN) infiltration was absent at 4 h post-LPS plus HI but significantly increased later (at 48 h), in the infarcted neocortical areas, compared to Ctl (Figure 4A, B). No histological change was noticed in the left hemisphere from animals exposed to LPS + HI (data not shown).

Early (4 h) and strong IL-1β response from neocortical and deep gray neurons, spreading to astrocytes of gray and white matter at 48 h, in rat forebrains after exposure to LPS plus HI.

**Figure 5:**
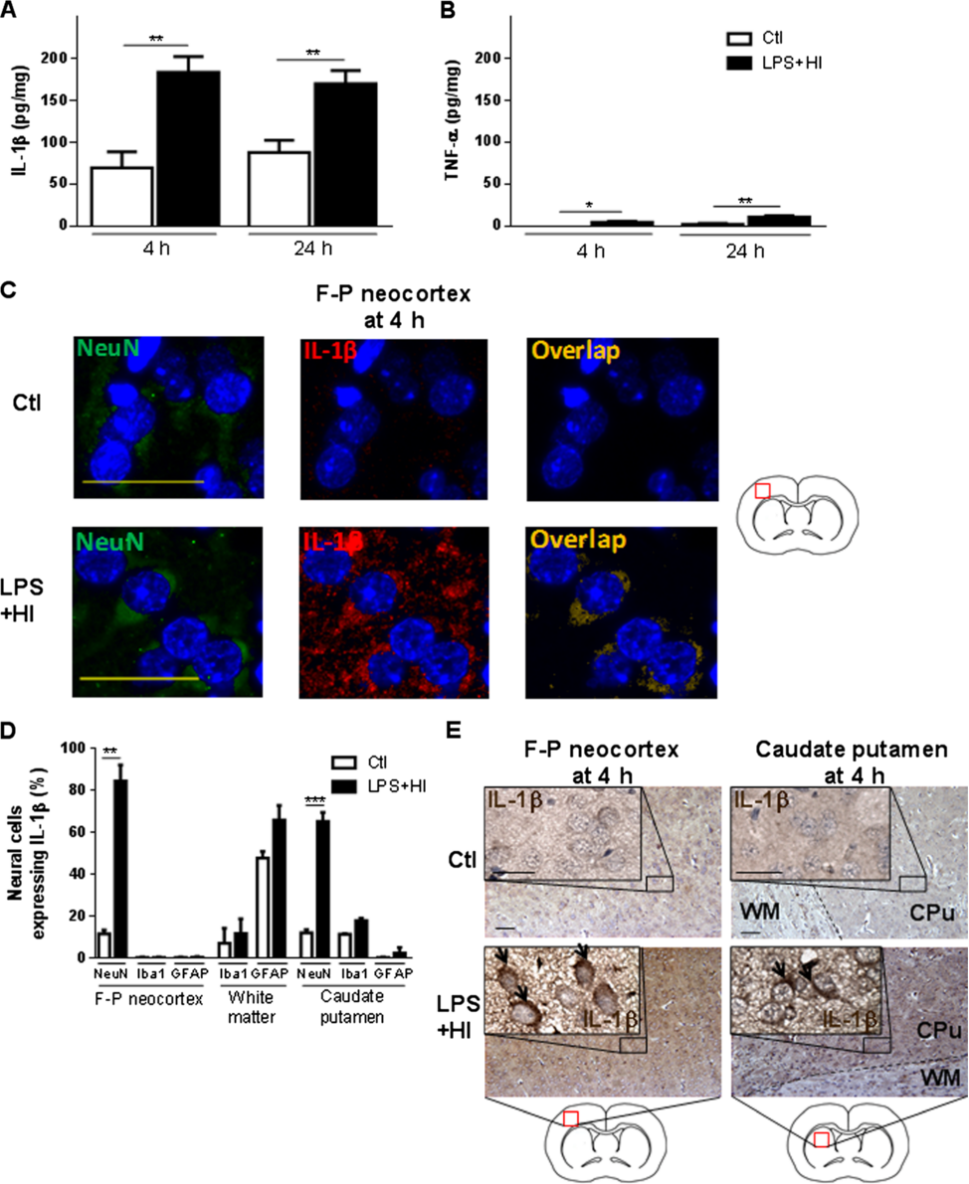
**Neuronal expression of IL-1β in the right hemisphere exposed to lipopolysaccharide (LPS) + hypoxia-ischemia (HI).** Mean ± standard error of the mean (SEM) (pg/mg) of IL-1β **(A)** and TNF-α **(B)**. Titrations were done by ELISA on total protein extracts from the right cerebral hemisphere exposed to LPS plus HI for 4 h to 24 h compared to control (Ctl). **(C)** Immunofluorescent staining of neuronal nuclei (NeuN) + IL-1β + cells in the fronto-parietal neocortex **(D)**. Mean ± SEM (%) of neural cells expressing Il-1β. **(E)** Immunohistochemical staining of IL-1β expression on neurons (arrows) from fronto-parietal neocortex and caudate putamen at 4 h post-LPS + HI compared to Ctl. Scale bar = 50 μm (4 to 6 pups per experimental condition). Analysis of variance with Newman-Keuls post-test; ^*^P ≤ 0.05, ^**^P ≤ 0.01. *h* hour, *F-P* Fronto-parietal, *WM* White matter, *CPu* Caudate putamen, *Iba1* Ionized calcium-binding adapter molecule 1, *GFAP* Glial fibrillary acidic protein.

**Figure 6:**
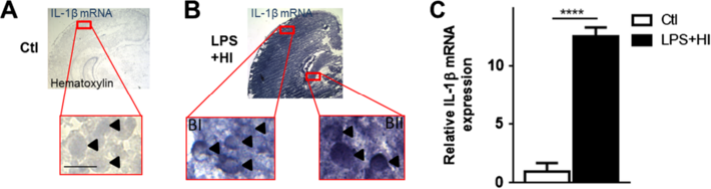
**IL-1β mRNA expression by in situ hybridization in the right hemisphere of lipopolysaccharide (LPS) + hypoxia-ischemia (HI)-exposed brains versus control (Ctl).** Absence of IL-1β mRNA detected in neurons from Ctl brains showing pure hematoxylin staining (arrowhead) **(A)**. High IL-1β mRNA expression (arrowhead) detected in neocortical **(BI)** and deep gray **(BII)** neurons 1 h after LPS + HI exposure **(B)**. Mean ± standard error of the mean IL-1β mRNA relative expression in gray matter of LPS + HI animals compared to Ctl **(C)** (3 animals per experimental condition). Scale bar = 10 μm. Unpaired *t*-test with Welch correction; ^****^P ≤ 0.0001.

**Figure 7:**
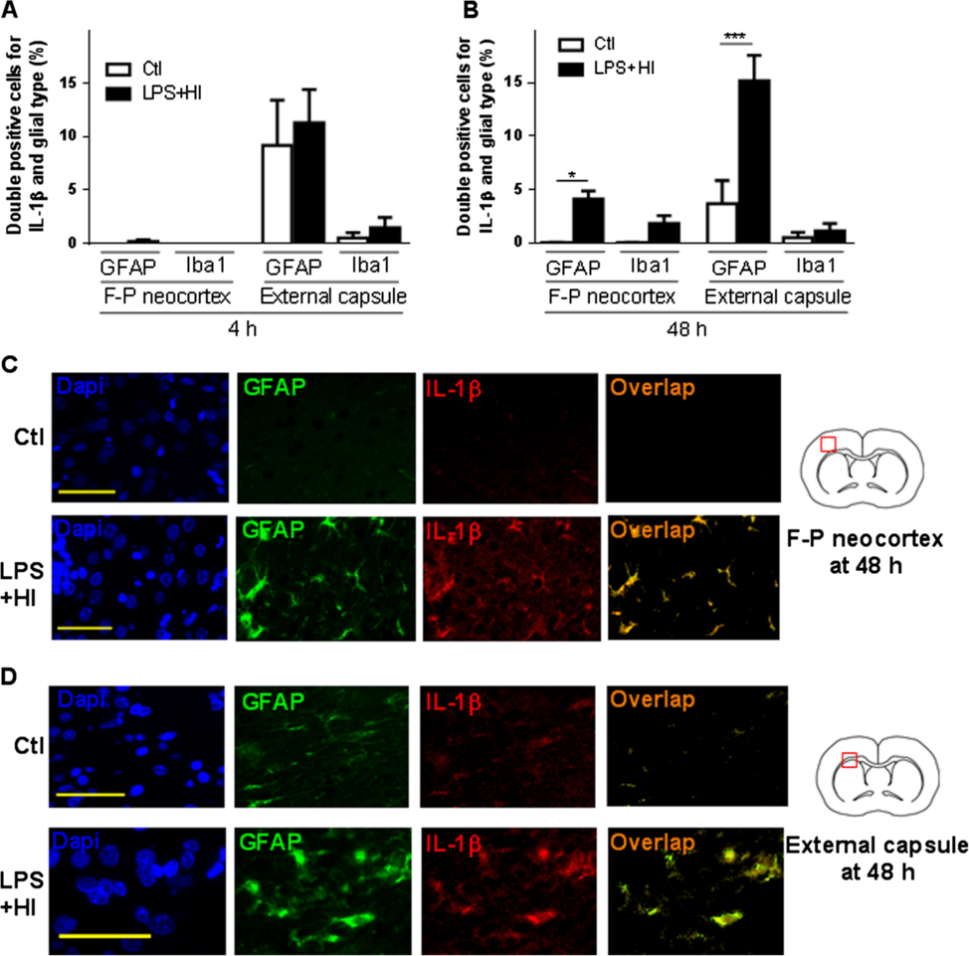
**Astroglial and macrophage/microglial expression of IL-1β in the right hemisphere exposed to lipopolysaccharide (LPS) + hypoxia-ischemia (HI).** Mean ± standard error of the mean ratio of Glial fibrillary acidic protein (GFAP) + IL-1β + or ionized calcium-binding adapter molecule 1 (Iba-1) + IL-1β + double-positive cells to total number of cells after LPS + HI exposure at 4 h **(A)** and 48 h **(B)** compared to control (Ctl). Immunofluorescent staining of GFAP + IL-1β + double-positive cells (overlap) in the fronto-parietal neocortex **(C)** and external capsule **(D)** at 48 h post-LPS + HI versus Ctl (3 to 4 pups per experimental condition). Scale bar = 50 μm. Analysis of variance with Newman-Keuls post-test; ^*^P ≤ 0.05, ^***^P ≤ 0.001. *h* hour, *F-P* Fronto-parietal, *DAPI* 4',6-diamidino-2-phenylindole.

ELISA results demonstrated an early increase in IL-1β after 4 h LPS + HI-exposure compared to Ctl (183.7 versus 69.5 pg/mg) (Figure 5A). On in situ labeling by IHC and IF, neuronal nuclei (NeuN)-positive neocortical and caudateputamen neurons were the only cell types in the brain with increased expression of IL-1β at 4 h after infarction (Figure 5C-E). This neuronal IL-1β expression was also assessed at the mRNA level 1 h post LPS + HI with in situ hybridization (Figure 6A, B). A 12-fold IL-1β mRNA expression was detected in all neurons from neocortical and deep gray areas subjected to damage, compared to Ctl (Figure 6C). IL-1β immunoreactivity was located in cellular bodies of fronto-parietal neurons from all six right neocortical layers of ongoing infarcted areas (Figure 5C). In fact, double staining on IF showed no significant difference in the proportion of IL-1β + GFAP + astrocytes and IL-1β + Iba-1+ microglia/macrophages between Ctl and LPS plus HI in the neocortex, white matter and striatum (Figure 5D, 7A). At 48 h, IL-1β expression was mainly present in neocortical neurons (data not shown). There was also a 4-fold increase in the percentage of IL-1β + GFAP + astrocytes - but not in IL-1β + Iba-1+ microglia/macrophage staining - within infarcted neocortical, white matter and caudate putamen areas of LPS + HI-exposed rats compared to Ctl (Figure 7B-D).

### Early and weak TNF-α production in microglial cells in forebrains of LPS plus HI exposed rats

**Figure 8:**
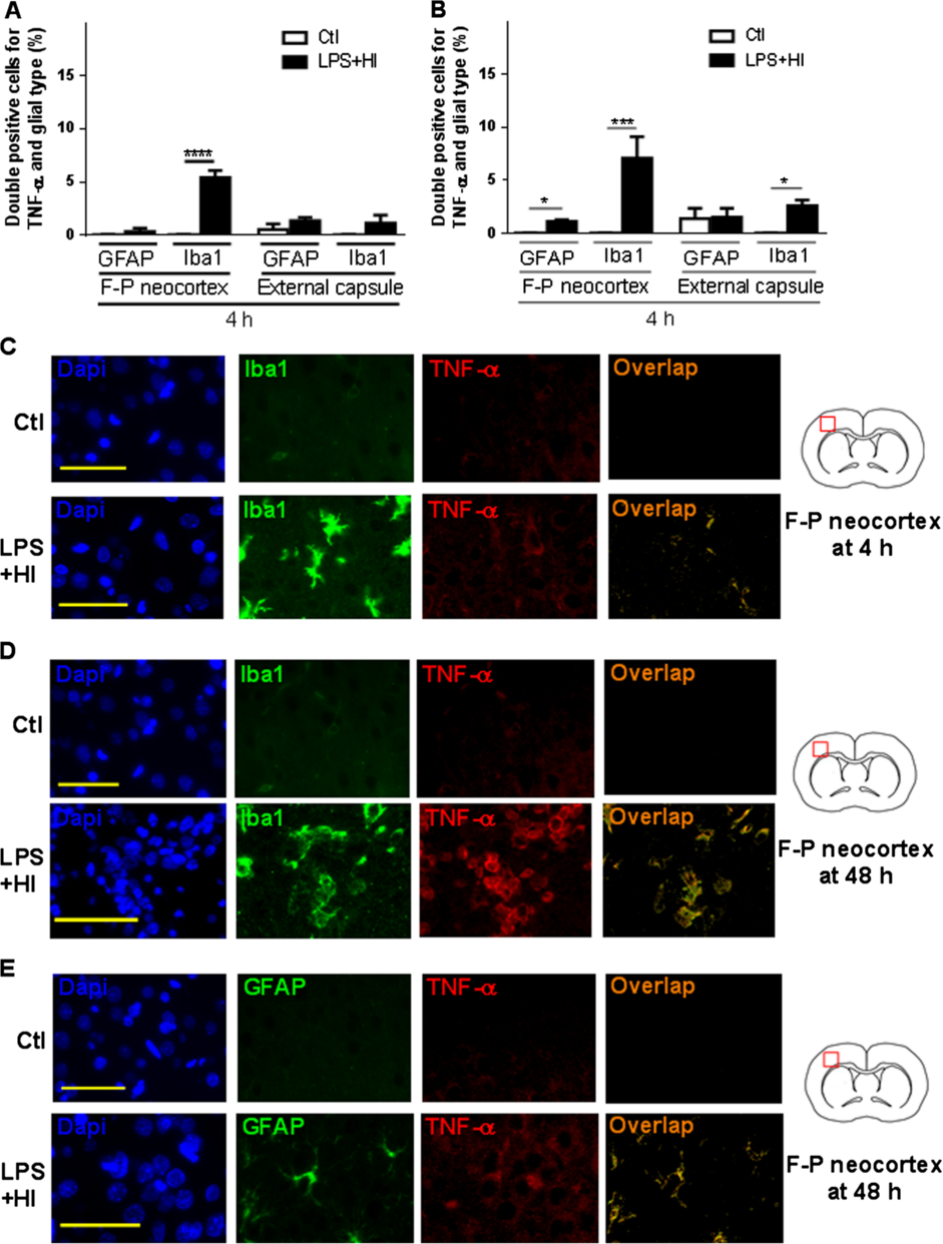
**Astroglial and macrophage/microglial expression of TNF-α in the right hemisphere exposed to lipopolysaccharide (LPS) + hypoxia-ischemia (HI).** Mean ± standard error of the mean ratio of Glial fibrillary acidic protein (GFAP) + TNF-α + or ionized calcium-binding adapter molecule 1 (Iba-1) + TNF-α + double-positive cells on total number of cells after 4 h **(A)** and 48 h **(B)**. Immunofluorescent (IF) staining of Iba-1+ TNF-α + double-positive (overlap) cells within fronto-parietal neocortex at 4 h **(C)** and 48 h **(D)** after LPS + HI versus control (Ctl). IF staining of GFAP + TNF-α + double-positive (overlap) cells within fronto-parietal neocortex at 48 h **(E)** after LPS + HI versus Ctl (3 to 4 pups per condition). Scale bar = 50 μm. Analysis of variance with Newman-Keuls post-test; ^*^P ≤ 0.05, ^***^P ≤ 0.001, ^****^P ≤ 0.0001. *h* hour, *F-P* Fronto-parietal, *DAPI*, 4',6-diamidino-2-phenylindole.

After LPS + HI exposure, a weak level of TNF-α expression (20-fold lower than IL-1β) was detected by ELISA at 4 h to 24 h post-HI (4 pg/mg at 4 h) compared to Ctl (0.02 pg/mg at 4 h) (Figure 5B). At 4 h post-HI, IF double-staining showed an increase in the number of TNF-α + Iba-1 + microglia/macrophage in the gray matter (neocortex and striatum) of LPS + HI-exposed rats (Figure 8A). At 48 h, an increase of TNF-α + Iba-1+ microglia/macrophages was noticed in both fronto-parietal lesioned gray and white matters, whereas the number of TNF-α + GFAP + cells were moderately increased only in the gray matter areas (Figure 8B, D, and E). There was no neuronal immunoreactivity for TNF-α at any time point (data not shown).

### Motor behavioral impairments in LPS + HI-exposed juvenile and adult rats

**Figure 9:**
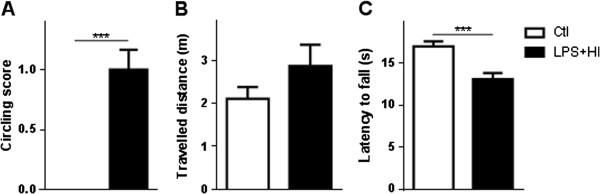
**Motor behavioral impairments of lipopolysaccharide (LPS) + hypoxia-ischemia (HI)-exposed rats between post-natal day (P)14 and P40. (A)** Mean ± standard error of the mean (SEM) travelled distance measured in the open-field test at P15 to P25 in LPS + HI-exposed pups compared to control (Ctl). **(B)** Mean ± SEM latency to fall of LPS + HI-exposed rats versus Ctl in the rotarod test at P30 to P40. **(C)** Mean ± SEM score of circling behavior at P14 in LPS + HI-exposed pups versus Ctl (10 to 14 pups per experimental condition). Unpaired *t*-test with Welch correction; ^***^P ≤ 0.001. *m* meter, *s* second.

**Figure 10:**
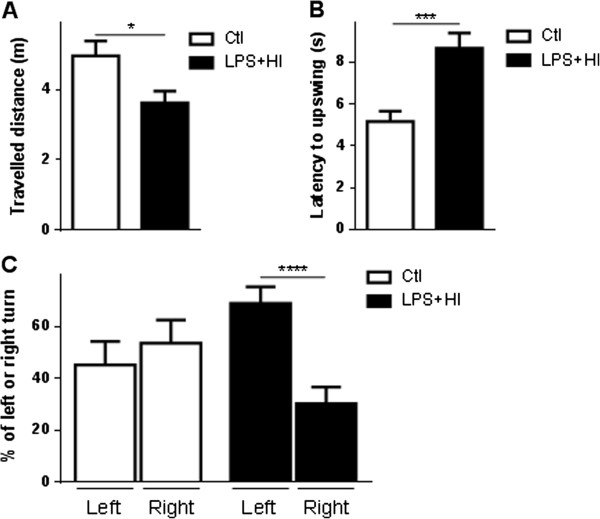
**Motor behavioral impairment of lipopolysaccharide (LPS) + hypoxia-ischemia (HI)-exposed rats at P100 to P120. (A)** Mean ± standard error of the mean (SEM) travelled distance measured in the open-field test in LPS + HI-exposed rats versus Ctl. **(B)** Mean ± SEM latency to upswing in the elevated-body-swing test in LPS + HI-exposed rats versus control (Ctl). **(C)** Mean ± SEM left or right turn in the turning-in-alley test in LPS + HI-exposed rats versus Ctl (8 to 10 rats per condition). Unpaired *t*-test with Welch correction; ^*^P ≤ 0.05, ^***^P ≤ 0.001, ^****^P ≤ 0.0001. *m* meter, *s* second.

Right or left circling behavior was transiently observed during the acute post-stroke phase at P13 to P14, in LPS + HI-exposed rats but not in Ctl (Figure 9A). The spontaneous locomotor activity recorded from P15 to P25 in the open-field test was similar in both Ctl and exposed rats (Figure 9B). The rotarod test at P30 showed decreased agility of LPS + HI-exposed rats compared to Ctl (Figure 9C). At the adult age (P100), the elevated body swing and open-field tests showed an overall decrease of spontaneous and forced motor activity in LPS + HI-treated rats compared to Ctl rats (Figure 10A, B). LPS + HI-exposed rats were significantly slower than Ctl to upswing (Figure 10B) and travelled a shorter distance (Figure 10A). Finally, a change in lateralization was observed with the turning-in-alley test for exposed rats: Ctl had a slight tendency to turn right (55%) whereas 70% of LPS plus HI-exposed rats turned left (Figure 10C).

### Effect of IL-1ra on LPS + HI induced brain damage

**Figure 11:**
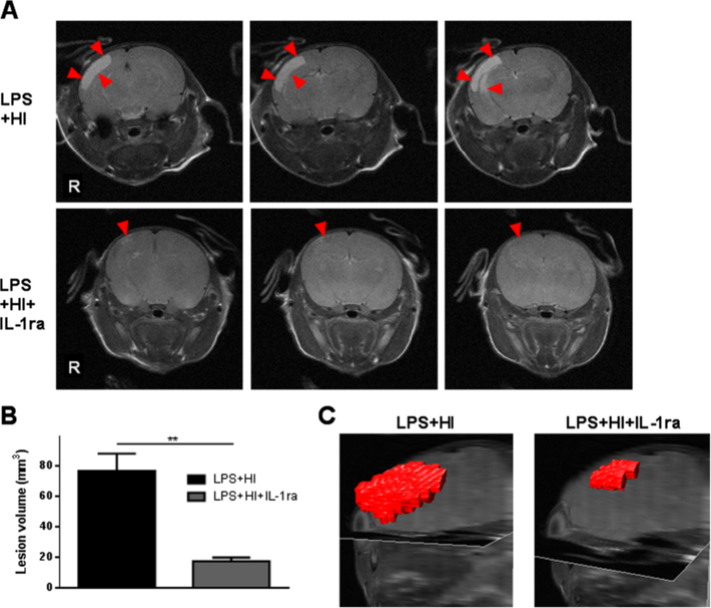
**Protective effect of IL-1 receptor antagonist (IL-1ra) on lipopolysaccharide (LPS) + hypoxia-ischemia (HI)-induced brain lesions. (A)** Magnetic resonance imaging of LPS + HI-exposed rat brains ± treatment with IL-1ra. Arrowheads show the areas of damage. No visible damage was observed in the left hemisphere. **(B)** Mean ± standard error of the mean lesion volume after LPS + HI exposition ± treatment with IL-1ra. **(C)** Three-dimensional reconstruction of the lesion after LPS + HI ± IL-1ra (5 to 6 rats per condition). Unpaired *t*-test with Welch correction; ^**^P ≤ 0.01. *R* right side, *mm*^3^ cubic millimeters.

Brain lesions were observed by MRI 8 days (P20) after LPS + HI. A wide hyper-signal area associated with the lesions was observed in the right cortex (Figure 11A, C). The treatment with IL-1ra decreased the extent of this area (Figure 11A, C). The total lesion volume decreased from 76.7 ± 11.7 mm^3^ to 17.5 ± 2.4 mm^3^ after IL-1ra treatment (Figure 11B).

## Discussion

The compounding effect of both LPS and HI on brain lesions had been previously reported in animal models of early and late preterm neonates [9,16]. This is the first report of neuropathological and behavioral outcomes of an animal model reproducing one of the most frequent pathological scenarios happening in the term human newborn context, namely pathogen exposure followed by subsequent HI [1,8]. The LPS + HI-induced brain lesions observed in this P12 rat model consist of large infarcts affecting frontal and parietal neocortex as well as adjacent subcortical white matter and caudate putamen. These lesions were observed at the same areas revealed by MRI. This neuropathological pattern is in sharp contrast with the one generated by LPS + HI-exposures at P1, that is*,* at a stage of brain development similar to a 26-week to 30-week premature human newborn [9,12,17]. In P1 pups, LPS + HI triggered a combination of radial columns and laminar foci of neocortical and caudate putamen neuronal death associated with patchy periventricular white matter loss, a typical pattern of the very premature human newborn [9]. At P7 (equivalent to the cerebral development of 32-week to 34-week gestation human newborns), LPS + HI-induced mixed patterns of P1 and P12 brain lesions by combining both foci of infarcts and selective columnar gray matter necrosis [6,7,16]. Thus, the differential impact of LPS + HI on the rat brain at relatively close developmental stages (P1 versus P7 versus P12) reflects the various profiles of brain damage observed in human newborns, stratified in their three principal stages of maturation, namely early preterm, late preterm or full-term neonates. The fact that brain lesions after LPS + HI-exposure at P12 are found in major cortical and subcortical motor centers likely accounts for the motor behavioral abnormalities that were demonstrated in this model, such as circling and decreased agility [18]. In other rodent neonatal models subjected to pure HI, transient hyperactivity has been mainly observed after HI brain injury induced in P6-9 rats. Nevertheless, eventual long-term behavioral impairments have been rarely investigated, except in the HI-exposed rabbit, which displayed symptoms of spasticity [19,20]. To our knowledge, our model provides the first demonstration of long-term motor impairments in rodents subjected to post-natal exposure to LPS + HI. These findings are relevant to cerebral palsy symptoms in humans [21]. However, the lack of spastic or dystonic symptoms, associated with the classic limitations of a rodent model (the level of neocortical development, and its distinct implication in motor control, compared to humans) should be mentioned [22].

Increased brain damage and mortality were observed in rats when HI was combined with LPS, compared to HI alone. One explanation could be that the transcription factor hypoxia-inducible factor (HIF)-1α may be activated by both HI and LPS stimulation. HIF-1α activation leads to pro-inflammatory cytokine up-regulation, thereby promoting cellular phagocytosis in anaerobic conditions [19,23]. Systemic LPS administration also induces an immune response in the brain through its direct interactions with Toll-like receptor 4 (TLR-4) [24]. Interestingly, in microglia and astrocytes, HI has been shown to indirectly activate the TLR-4 pathway through endogenous ligands, such as heat-shock proteins [25]. Activation of TLR-4 triggers the Nuclear factor-κB (NF-κB) pathway, which is also activated by IL-1β and TNF-α [26]. NF-κB and HIF-1α are pro-inflammatory transcription factors that operate conjointly within microglial and astroglial cells under the combined activation with both LPS and HI. Pro-IL-1β has to be cleaved by a macromolecular complex called inflammasome so that the IL-1β secretion occurs [27]. Most of the central nervous system cells, including neurons, astrocytes, and microglia, express cellular machinery to assemble inflammasome and therefore are able of IL-1β secretion [28]. The exact nature of the inflammasome assembling and triggering stimuli is unclear, but different pathogen-associated molecular patterns (PAMPs) and damage-associated molecular patterns (DAMPs) initiate inflammasome-dependent IL-1β processing [27]. It depends on the presence of different nod-like receptors (NLRs) that are responsible for sensing intracellular milieu and triggering inflammasome assembly [27]. It has been shown that the inflammasome is activated after brain injury [28]. NLRP1 and NLRP3 inflammasomes are present in neurons and microglia respectively [28]. They both trigger the activation of pro-IL-1β and are activated by different PAMPs, such as LPS, and DAMPs that are endogeneous molecules released after cellular damage. Exposure to LPS and HI leads to the production of DAMPs and, when combined, could lead to an exacerbated activation and secretion of IL-1β, possibly through NLRP1 inflammasome activation [28]. Such ability of neurons to produce IL-1β mRNA and protein has also been shown by previous studies using either rodents subjected to HI, traumatic, and excitotoxic brain injuries, or samples of lesioned human perinatal brains [29-31]. Thus, exposure to LPS + HI, at this specific term-like stage of brain development, might be responsible for neuronal self-injuries via IL-1β production through well-known neurotoxic mechanisms, such as IL-1β-induced nitric oxide production, or IL-1β exacerbation of excitotoxic damage [32]. Consistent with this hypothesis, blocking the signaling pathway of IL-1β in the presence of IL-1ra, decreased the extent of brain injury in LPS + HI-exposed rats.

## Conclusion

Overall, after LPS + HI, IL-1β is expressed mainly by neurons at 4 h post-aggression and, later, by neurons and microglial cells at 48 h. This expression exacerbates neuroinflammation and cerebral injury resulting in delayed onset of profound motor impairments. Interfering with IL-1β signaling decreased the extent of brain lesions.

## Abbreviations

ANOVA: analysis of variance; BCIP: (5-bromo-4-chloro-1H-indol-3-yl) dihydrogen phosphate; Ctl: Control group; DAMP: Damage-associated molecular pattern; DAPI: 4',6-diamidino-2-phenylindole; ELISA: Enzyme-linked immunosorbent assay; FOV: Field of view; G: Gestational day; GFAP: Glial fibrillary acidic protein; H&E: Hematoxylin-eosin; HI: Hypoxia-ischemia; HIF: Hypoxia-inducible factor; Iba1: Ionized calcium-binding adapter molecule 1; IF: Immunofluorescence; IHC: Immunohistochemistry; IL: Interleukin; IL-1ra: Interleukin-1 receptor antagonist; LPS: Lipopolysaccharide; MRI: Magnetic resonance imaging; NBT: Nitro blue tetrazolium chloride; NeuN: Neuronal nuclei; NF-κB: Nuclear factor-κB; NLR: Nod-like receptor; P1: Post-natal day 1; PAMP: Pathogen-associated molecular pattern; PFA: Paraformaldehyde; PMN: Polymorphonuclear neutrophil; SEM: Standard error of the mean; TEA: Triethanolamine; TLR: Toll-like receptor; TNF: Tumor necrosis factor.

## Competing interests

The authors declare that they have no competing interests.

## Authors’ contributions

AS, KL and MEB carried out the experiments and performed the statistical analyses. AS and KL drafted the manuscript. ML was responsible for the MRI section of the study. GS conceived the study and coordinated the project. DG, MEB and GS helped to draft the manuscript. All authors read and approved the final manuscript.
